# Oral health care among dialysis patients in France and impact on survival

**DOI:** 10.1007/s40620-025-02307-4

**Published:** 2025-05-30

**Authors:** Linda Jordane Dondjio Jemele, Marie Buzzi, Orly Petipa Nga, Cécile Couchoud

**Affiliations:** 1 Sorbonne Université, Paris, France; 2 Université de Lorraine, Inserm, INSPIIRE, 54000 Nancy, France; 3 CHRU-Nancy, INSERM, Université de Lorraine, CIC, Epidémiologie Clinique, Nancy, France; 4 Université Paris-Saclay, UVSQ, Inserm, CESP, 94807 Villejuif, France; 5 Faculté de Santé, Université Paris Cité, UFR d’odontologie, Montrouge, France; 6 REIN Registry, Agence de la Biomédecine, La Plaine Saint-Denis, Paris, France

**Keywords:** Care use, Oral care, Dialysis, Survival

## Abstract

**Background:**

Oral diseases have been shown to be risk factors of Chronic Kidney Disease (CKD) progression and may also be associated with poorer survival. The aim of this study was to describe oral care of dialysis patients in France and to assess its impact on their survival.

**Methods:**

We conducted a retrospective cohort study including all patients on dialysis in France between 2015 and 2020. Data were collected from the French REIN registry and matched with the National Health Data System. Factors associated with the probability of receiving oral care treatments were explored using a logistic regression model. The survival of incident dialysis patients was modeled using a Cox model.

**Results:**

Among the 101,942 prevalent patients included in our sample, 32.5% received oral care treatment over the 6-year study period. Average annual adoption was 18.7% (versus 43% in the general French population) with regional variations. Male gender, overweight, dialysis treatment < 3 and > 6 years, and being on the transplant waiting list were associated with greater oral care treatment. Oral care treatment was associated with a lower risk of death (weighted hazard ratio (HR) 0.53 [0.51–0.55], adjusted HR 0.50 [0.48–0.52] 95% confidence interval (CI)).

**Conclusion:**

Dialysis patients in France undergo a low level of oral care treatments. Patients' characteristics and regional practices appear to influence this. Oral care treatments seem to have a positive impact on survival.

**Graphical abstract:**

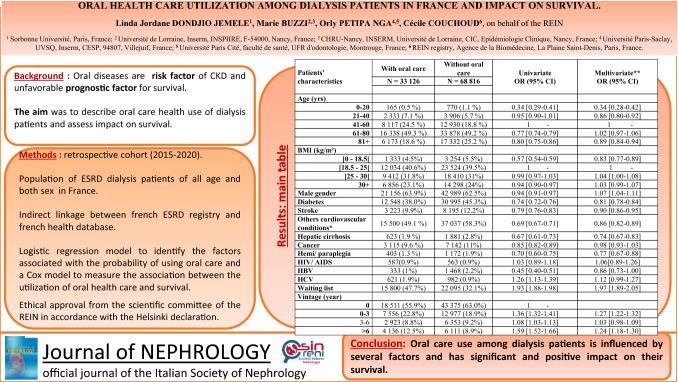

**Supplementary Information:**

The online version contains supplementary material available at 10.1007/s40620-025-02307-4.

## Introduction

A number of disorders of the oral sphere have been described in patients with Chronic Kidney Disease (CKD) [1]. Such disorders include mucosal changes (pallor, uremic stomatitis, cheilitis, ulcerations, ecchymoses, hyperplastic lesions, lichenoid lesions, macroglossia), dental affections (enamel hypoplasia, ultrastructural changes in dentine, pulpal calcifications, erosions, dyschromias, malocclusions), periodontal disorders (dental mobility, bleeding, gingivitis, periodontitis), and salivary changes (xerostomia, dysgeusia, halitosis, increased salivary gland volumes) [2]. Increase of oral pH slows down plaque formation and lowers the caries index in these patients, while a rise in salivary urea concentration accelerates tartar formation and thus the onset of periodontal disease. Osteodystrophy of the mandibular, maxillary, and alveolar bones might also appear as a manifestation of mineral-bone disorders [3]. Because of their often-precarious health, dialysis patients have a low level of oral hygiene due to frequent multiple edentulism, unsuitable dentures (due to osteodystrophy for example) and a low masticatory coefficient [4]. Oropharyngeal candidiasis, often due to polymedication, may also be present, leading to undernutrition, which has a significant impact on quality of life and prognosis [5].

Recent studies have presented oral diseases not only as manifestations or consequences of CKD, but also as potential risk and/or prognostic factors [6–8]. A bidirectional relationship exists between CKD and oral diseases, particularly periodontitis. This relationship is characterized by common pathophysiological and dynamic biological interconnections, including systemic inflammation, endothelial dysfunction, and oxidative stress imbalance. The oral microbiota, particularly the periodontal microbiota, is considered a significant modifier of chronic kidney disease. Furthermore, individuals with long-term periodontitis appear to be at high risk of declining renal function over time, and patients with CKD and periodontitis are at risk of an unfavorable course, with increased morbidity and mortality [9]. Thus, poor oral health in a CKD patient should be a warning sign.

The main aim of this study was therefore to assess the prevalence of oral health care treatments by patients undergoing chronic dialysis in France between 2015 and 2020, and to identify factors associated with it. A secondary aim was to assess the impact of oral health care treatments on survival in incident patients starting chronic dialysis.

## Methods

We conducted a retrospective cohort study over a 6-year period (01.01.2015–31.12.2020) based on two national databases.

The REIN registry (Epidemiology and Information Network in Nephrology) is a national register supported institutionally by the French Biomedicine Agency [10–12]. Its objective is the epidemiological follow-up of all patients with kidney failure treated by kidney replacement therapy (KRT) in France. Clinical, demographic, and laboratory data are collected at the start of KRT along with dialysis modalities, and are updated annually. Events such as death, transfer from dialysis center, withdrawal from dialysis, placement on a transplant waiting list, and kidney transplantation (from living or deceased donors) are systematically reported in real-time.

The SNDS (National Health Data System) contains detailed information on medical care for the population of France [13].

The REIN-SNDS merged database was built up using indirect, deterministic matching on six variables: patient’s age, sex, date and place of KRT initiation, place of residence and date of death [14].

### Population

Our study included male and female kidney failure patients of all ages in France, who had benefited from at least one chronic dialysis session between 01.01.2015 and 31.12.2020. Transplant recipients without dialysis between 2015 and 2020, as well as participants who could not be matched between the two databases (SNDS and REIN) were not included.

### Variables

We used the following variables available in the REIN registry: socio-demographic data (gender, age, region of treatment), comorbidities (diabetes, stroke/transient ischemic attack, coronary disease, heart failure, heart rhythm disorders, lower limb arteritis, aortic aneurysm, cancer, HIV/AIDS, hepatitis B and C, cirrhosis, hemi/paraplegia), Body Mass Index (BMI) and events of interest (date of first dialysis, date of renal transplant, date of registration on the renal transplant waiting list, date of death).

The data retrieved from the SNDS were all reimbursements for oral care treatments (date and type of care). The medical procedure classification codes used in the database were grouped into 8 categories (conservative, prosthetic, prophylactic, endodontic, periodontal, implant and orthodontic treatments and consultation/prevention sessions).

### Statistical analysis

Descriptive data analysis was computed for all variables of interest.

Regional prevalence of oral care treatment was obtained by dividing the number of dialysis patients having benefited from oral care treatments over the study period by the total number of dialysis patients present in the region over the same period. The regional distribution of oral care treatments was represented on a map generated by MAGRIT software version 2.1.0.

Patients receiving and not receiving oral care treatments over the study period were compared and a multivariate logistic regression model was employed to identify the factors associated with the probability of receiving oral care treatment. The results were expressed as Odds Ratios (ORs) with their 95% confidence intervals (CIs).

Survival after dialysis initiation was explored only in the subgroup of incident patients (i.e., patients who had initiated dialysis during the study period) and the endpoint was defined at 6-year survival. Exposure was defined as the utilization of oral health care services, measured dichotomously as either present or absent, without consideration of the specific nature of the care provided. Kaplan–Meier curves were plotted according to the 2 groups (receiving and not receiving oral care treatments). To avoid any immortal time bias, all patients were considered as non-recipients of oral care treatment at inclusion; patients who had benefited from oral care treatments during the study period were considered as recipients of oral care from the first date of reimbursement for an oral care procedure.

The association between oral care treatments and death was analyzed via Cox proportional hazards regressions. To overcome some indication bias, an inverse probability treatment weight (IPTW) of receiving oral care treatment was introduced in the model (weighted model), and secondly, covariates were added to the model (model adjusted for age, sex, comorbidities and region of treatment). The stabilized weights were estimated by the previous logistic regression model including age, comorbidities and region of treatment. Patients were censored at renal transplantation or dialysis withdrawal.

Date of first oral care treatment and date of registration on the transplant waiting list were introduced as time-dependent variables to overcome immortality bias. Results are expressed as HRs with their 95% CIs.

Missing data were not imputed.

Statistical analyses were performed with SAS software, version 9.4 (SAS Institute, Inc., Cary, NC, USA).

## Results

A total of 112,554 patients registered in the REIN registry had at least one non-acute dialysis session between January 01, 2015 and December 31, 2020. Among them, 101,942 could be matched with patients from the SNDS database and constituted our study sample. Among this sample, 61,866 were incident dialysis patients (i.e., patients who had initiated dialysis during the study period).

Patients were mostly male, with a sex ratio of 1:7. Mean age was 67.2 ± 15.9 years, with extremes of 0 and 104 years.

### Oral health care treatment and associated factors

In our population of prevalent patients on dialysis (i.e., whole sample), 32.5% had benefited from oral health care treatments between January 1, 2015 and December 31, 2020.

Male gender, being overweight, duration of dialysis treatment < 3 years or > 6 years and registration on the transplant waiting list were associated with a higher probability of receiving oral care treatments.

In all age groups except the age group 61–80 years (vs. 41–60), being underweight, having comorbidities (diabetes, antecedent of stroke, other cardiovascular conditions, cirrhosis, hemi/paraplegia) were associated with a lower probability of undergoing dental care treatments (Table 1) .

**Table 1:** Characteristics of REIN—SNDS dialysis matched patients receiving and not receiving oral care treatments

Patients' characteristics	Receiving oral care treatment	Not receiving oral care treatment	UnivariateOR (95% CI)	Multivariate** OR (95% CI)
	*N* = 33,126	*N* = 68,816		
Age (yrs)				
0–20	165 (0.5%)	770 (1.1%)	0.34 [0.29–0.41]	0.34 [0.28–0.42]
21–40	2333 (7.1%)	3906 (5.7%)	0.95 [0.90–1.01]	0.86 [0.80–0.92]
41–60	8117 (24.5%)	12,930 (18.8%)	1 -	1 -
61–80	16,338 (49.3%)	33,878 (49.2%)	0.77 [0.74–0.79]	1.02 [0.97–1.06]
81 +	6173 (18.6%)	17,332 (25.2%)	0.80 [0.75–0.86]	0.89 [0.84–0.94]
BMI (kg/m^2^)				
[0–18.5]	1333 (4.5%)	3254 (5.5%)	0.57 [0.54–0.59]	0.83 [0.77–0.89]
[18.5–25]	12,034 (40.6%)	23,524 (39.5%)	1 -	1 -
[25–30]	9412 (31.8%)	18,410 (31%)	0.99 [0.97–1.03]	1.04 [1.00–1.08]
30 +	6856 (23.1%)	14,298 (24%)	0.94 [0.90–0.97]	1.03 [0.99–1.07]
Male gender	21,156 (63.9%)	42,989 (62.5%)	0.94 [0.91–0.97]	1.07 [1.04–1.11]
Diabetes	12,548 (38.0%)	30,995 (45.3%)	0.74 [0.72–0.76]	0.81 [0.78–0.84]
Stroke	3223 (9.9%)	8195 (12.2%)	0.79 [0.76–0.83]	0.90 [0.86–0.95]
Others cardiovascular conditions*	15,500 (49.1%)	37,037 (58.3%)	0.69 [0.67–0.71]	0.86 [0.82–0.89]
Hepatic cirrhosis	623 (1.9%)	1881 (2.8%)	0.67 [0.61–0.73]	0.74 [0.67–0.83]
Cancer	3115 (9.6%)	7142 (11%)	0.85 [0.82–0.89]	0.98 [0.93–1.03]
Hemi/paraplegia	403 (1.3%)	1172 (1.9%)	0.70 [0.60–0.75]	0.77 [0.67–0.88]
HIV/AIDS	587(0.9%)	563 (0.9%)	1.03 [0.89–1.18]	1.06[0.89–1.26]
HBV	333 (1%)	1468 (2.2%)	0.45 [0.40–0.51]	0.86 [0.73–1.00]
HCV	621 (1.9%)	982 (0.9%)	1.26 [1.13–1.39]	1.12 [0.99–1.27]
Waiting list	15,800 (47.7%)	22,095 (32.1%)	1.93 [1.88–1.98]	1.97 [1.89–2.05]
Vintage (year)				
0	18,511 (55.9%)	43,375 (63.0%)	1 -	
0–3	7556 (22.8%)	12,977 (18.9%)	1.36 [1.32–1.41]	1.27 [1.22–1.32]
3–6	2923 (8.8%)	6353 (9.2%)	1.08 [1.03–1.13]	1.03 [0.98–1.09]
> 6	4136 (12.5%)	6111 (8.9%)	1.59 [1.52–1.66]	1.24 [1.18–1.30]

*At least one of the following: coronary diseases, heart failure, rhythm disorders, lower limb arteritis, aortic aneurysm

**Adjusted for patients’ characteristics and region of treatment

### Regional distribution of oral care treatments

Prevalence of oral care treatments exhibited significant regional variations independently of patient characteristics (Supplementary Fig. 1). The Ile-de-France region documented the highest number of treatments over a six-year period (5500), with a utilization rate of 30%. In five regions less than 30% of dialysis patients received oral care treatment over a six-year period, reaching 15% in Mayotte. The region of Reunion demonstrated the highest adoption of oral care treatment, with an estimated rate of 39%. The observed variations among regions were found to be statistically significant.

### Temporal evolution of oral care treatment

A total of 186,440 treatments were administered between 2015 and 2020, with a peak in 2019, with 33,730 oral treatments. The peak number of dialysis patients was observed in 2017, with 51,208 patients. The lowest figures were in 2020 with 29,375 oral care treatments involving 8181 patients (Supplementary Fig. 2). Annual oral care treatment prevalence from 2015 to 2020 was 18.9%, 19.2%, 19%, 19.1%, 19.5% and 16.4%, with an average of 18.7%.

### Type of oral care treatments

**Fig. 1:**
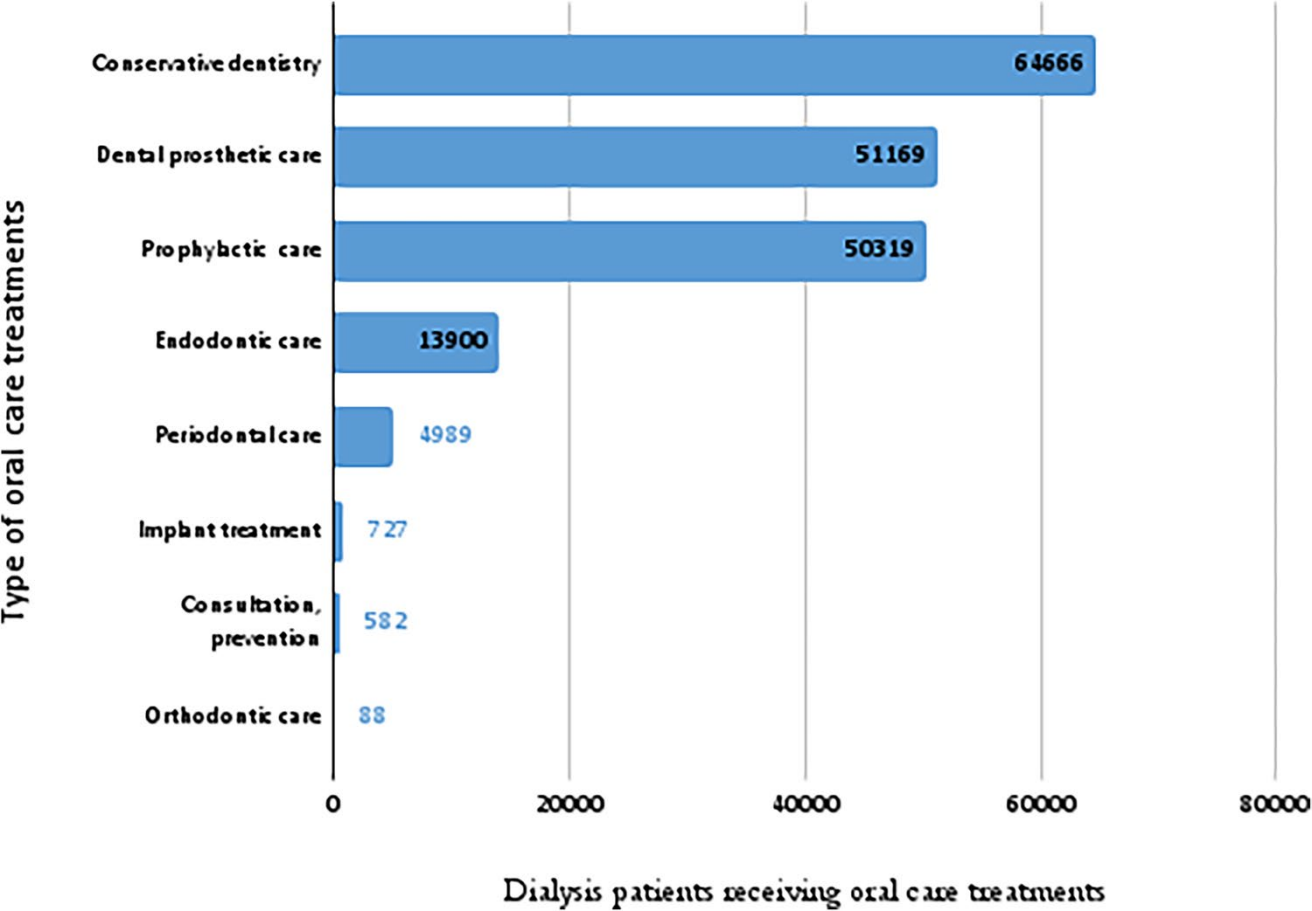
Type of oral care treatments dialysis patients received in France from 2015 to 2020

Dental restorations using composite or amalgam were the most common treatments dialysis patients received (35%), while orthodontic treatment was by far the least common (0.04%) (Fig. 1).

### Impact of oral care treatment on survival

**Fig. 2:**
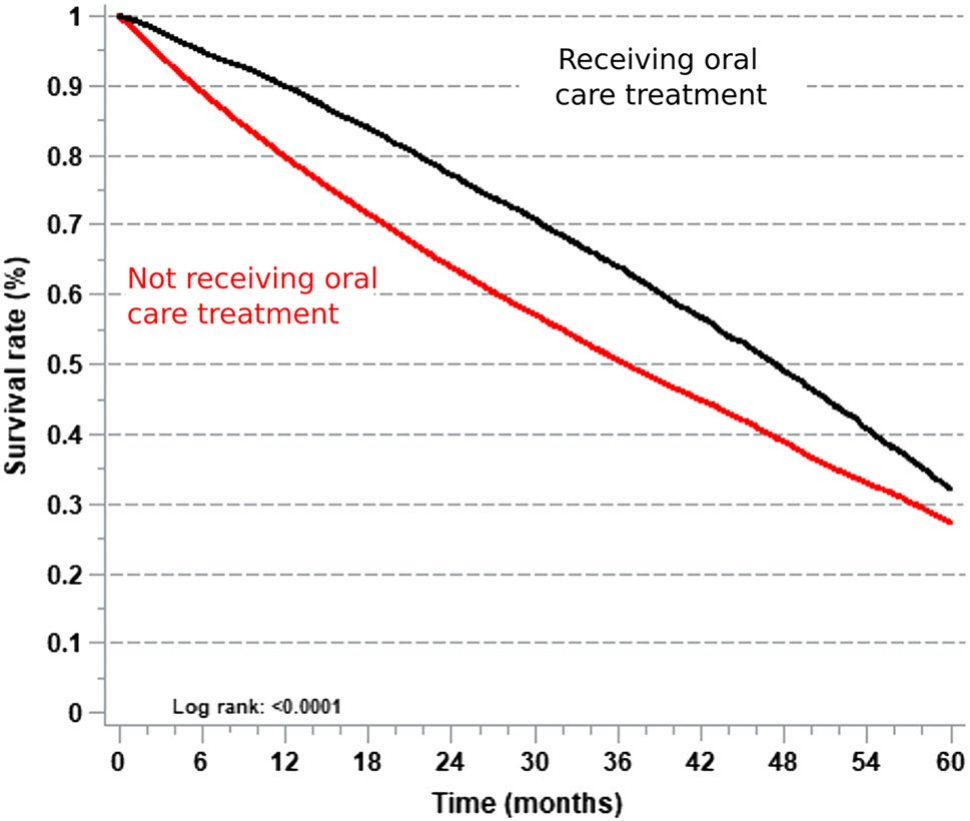
Kaplan–Meier survival curves of dialysis patients receiving and not receiving oral care treatments

Among the 61,866 incident dialysis patients, 18,511 received oral care treatment and 19,597 died during the study period. Mean follow-up was 38 ± 0.1 months among the non-recipients of oral care treatment and 44.1 ± 0.3 months in the recipients of oral care treatment. The proportion of patients who died during the study period was higher in the group not receiving oral care treatments (Fig. 2).

**Fig. 3:**
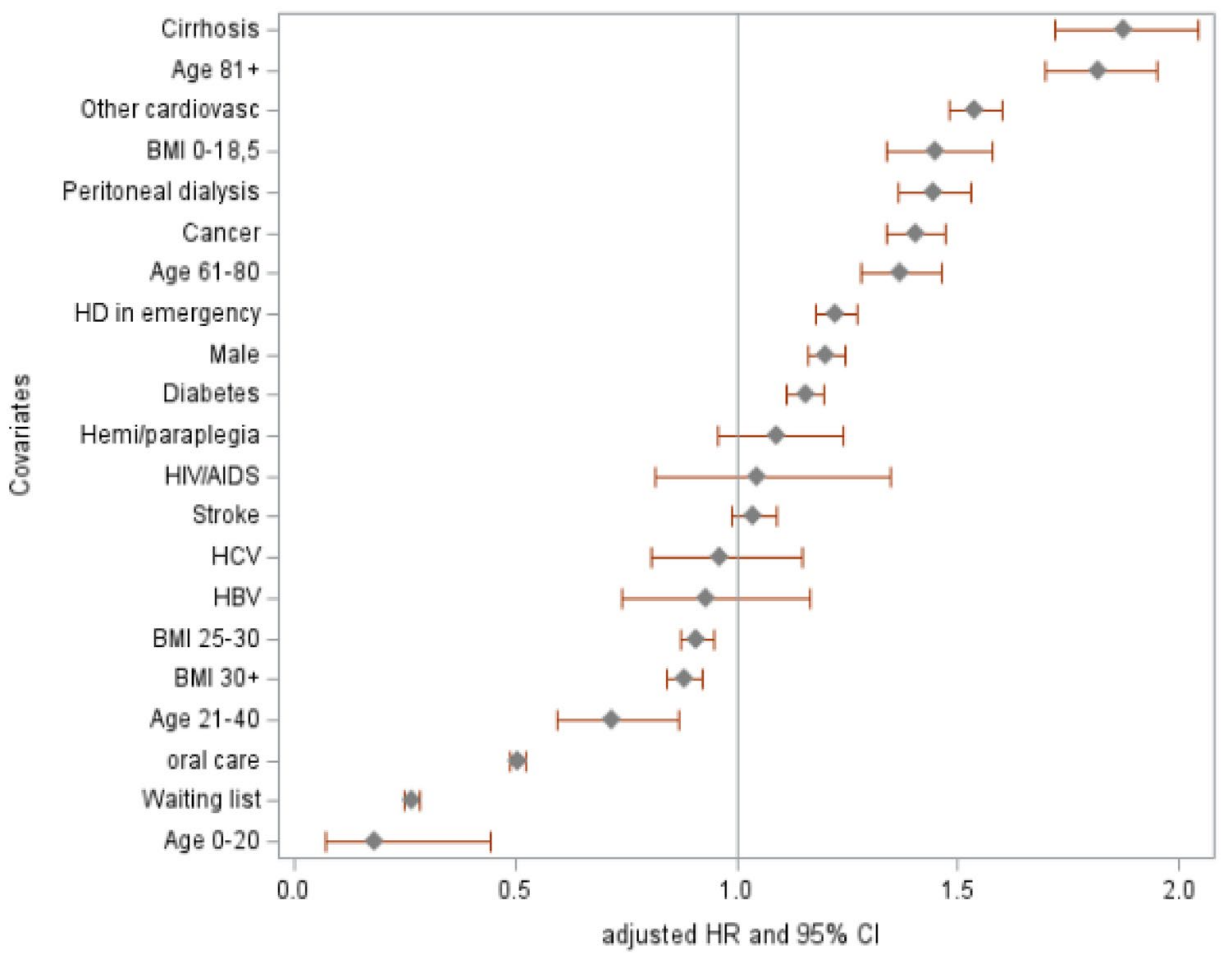
Forest plot of adjusted Hazard Ratios of dialysis patients receiving and not receiving oral care treatment

Oral care was associated with an approximately 50% reduction in mortality: weighted HR 0.53 [95% CI 0.51–0.55], adjusted HR 0.50 [0.48–0.52] (Supplementary Table 1). Being on the transplant waiting list, age < 40 years, and overweight/obese were statistically associated with better survival (Fig. 3). However, factors including hemodialysis in emergency, peritoneal dialysis, male gender, diabetes, cancer, liver cirrhosis, BMI less than 18.5, age over 40 years and cardiovascular comorbidities, were found to be statistically associated with poorer survival.

## Discussion

Our study showed that dialysis patients undergo few oral care treatments.

Grubbs et al. showed that CKD patients from an American cohort were 25% less likely to undergo a dental visit than non-CKD patients, after adjustment for confounding factors [15]. In Japan, Kaneko et al. found that kidney failure patients were also much less likely to visit the dentist than CKD patients [16].

The ORAL-D study, which examined the relationship between periodontitis and mortality in hemodialysis patients, was conducted in seven countries, including France. The findings of Frantzen et al. indicated that patients who received a dental consultation within six months exhibited a 20% improved survival rate compared to those who did not [17].

Systematized oral care treatments with regular check-ups would therefore be an important factor for reducing morbidity and early mortality in CKD patients.

In France, the average annual prevalence of oral care treatment by kidney failure patients from 2015 to 2020 was 18.7%. This value is well below the annual recourse by the general population in France, which was estimated at 43% for all ages combined in 2014 [18]. This could be explained by the fact that kidney failure patients on dialysis are often frail, highly hospitalized and may not be aware of the importance of maintaining good oral health. However, analyzing oral care treatment is complex, as it may reflect both good and poor oral health among dialysis patients. Low adoption of oral care treatment may reflect the absence of need and therefore good oral health, on one hand, or a problem of access to such care or a lack of interest by the patient and the care team on the other hand.

Our description of the evolution of oral care treatment over time showed a decline in the number of administered oral care treatments in 2020. The health crisis caused by the COVID-19 pandemic is an obvious explanation for this finding, with an overall drop in the use of healthcare and a decline in visits to healthcare establishments [19].

Geographical variations of oral care treatments were also described in the general population [20]. The non-uniform dialysis patients’ case-mix across regions, but also differences in terms of access to health care, could explain these differences [21]. Future studies are needed to explore this topic further.

Conservative, prosthetic and prophylactic care alone accounted for almost 90% of the oral health care treatments provided to the patients in our study. This is consistent with the treatments most frequently provided in dental practice [22], especially in a population of patients with rapidly deteriorating periodontal status and frequent dental disease and loss. It seems therefore likely that the adoption of oral care treatments (or lack thereof) would be an indicator of poor oral health in these patients.

In our study, we found that the probability of recourse to the dentist was lowest in younger patients, unlike Grubbs et al., who found a decrease in the probability of recourse with age [15]. This difference may be partly explained by the fact that we included minors (aged 0–20) in our study. The latter, having a better oral health capital, will tend to consult less. But still, their access to care remains conditioned by third parties.

Dialysis patients on the transplant waiting list were 2 times more likely to seek oral care treatment than non-registered patients. Recommendations aiming to remove all potential sources of infection in patients awaiting transplantation encourage them to seek dental care [23]. This was also reflected in our survival analysis, as these patients had better survival than the others. This further emphasizes the fact that the under-adoption of oral care treatment in dialysis patients may be at least in part related to a lack of information about the influence of oral health on the morbidity and mortality of their pathology.

To the best of our knowledge, this is the first study to have investigated oral health care treatment by chronic dialysis patients in France, and its impact on their survival. It presents several strengths. First, it is based on reliable and rather exhaustive national data. Moreover, the study was carried out over a fairly recent period. However, the absence of clinical data on patients' health status was a significant limitation, as this information would have supported the association between kidney and oral health, as has already been detailed in the literature. The inclusion of such clinical data would also have enabled a more precise assessment of the cause-effect relationship and its impact on mortality, as well as demonstrating that patients receiving little oral care treatment had worse clinical conditions. Access to dental care, oral hygiene, oral health knowledge, lifestyle, socio-economic factors and level of education were also important factors that could not be collected in the present study. Despite the potential for this retrospective study design to be perceived as a limitation, it is imperative to recognize that it represents the optimal approach for the accumulation of data on extensive patient populations. Furthermore, the two databases utilized employ systematic data collection methodologies, ensuring the integrity and reliability of the collected information. This study's findings are relevant to other countries as well, given the pertinence of the clinical issue. Furthermore, there are countries with local databases or insurance data that can be used for analogous studies.

## Conclusion

Our study, which investigated the adoption of oral health care treatments by dialysis patients with kidney failure in France between 2015 and 2020, revealed that oral health care treatment is low compared to the general population.

The adoption of oral care treatments was shown to be influenced by several factors such as age, BMI, comorbidities, region of dialysis, and registration on the transplant waiting list. Oral care treatments also appeared to have a significant and positive impact on the survival of dialysis patients.

The results of this study show that improvement in the oral care of dialysis patients in France is possible. Health authorities should take this into account by promoting close collaboration between patients, nephrologists and dental surgeons, and raising awareness of this issue.

## Supplementary Information

Below is the link to the electronic supplementary material.

Supplementary file1 (DOCX 105 kb)

## Data Availability

Data used for this research were extracted from the REIN registry, coordinated and supported by the French Biomedecine Agency. The access to national data is regulated by a scientific committee of the French Biomedecine Agency which analyzes each request, and so cannot be made publicly available due to legal restrictions.As well, access to the data of National Health database cannot be made publicly available due to legal restrictions without requesting individual authorization.

## List of Abbreviations

CKD: Chronic kidney disease
GFR: Glomerular filtration rate
ESKD: End stage kidney disease
REIN: Nephrology Epidemiology and Information Network
SNDS: National Health Data System
KRT: Kidney replacement therapy
BMI: Body mass index
HD: Hemodialysis
TIA: Transient ischemic accident
HIV: Human immunodeficiency virus
AIDS: Acquired immunodeficiency syndrome
HBV: Hepatitis B virus
HCV: Hepatitis C virus
IPTW: Inverse probability treatment weight

## Declarations

### Human and animals rights

The author state that this research was conducted in accordance to the Helsinki declaration of 1975 and its revision.
