# Supplementary material for: Oral health care among dialysis patients in France and impact on survival
Source: J Nephrol. 2025 May 30;38(8):2213–20. doi: 10.1007/s40620-025-02307-4 (PMC12630281; doi:10.1007/s40620-025-02307-4)
Supplement: Supplementary file 1 — Supplementary file1 (DOCX 105 kb) [file 40620_2025_2307_MOESM1_ESM.docx]

**ORAL HEALTH CARE USE AMONG DIALYSIS PATIENTS IN FRANCE AND IMPACT ON SURVIVAL**

**Linda Jordane DONDJIO JEMELE^1^, Marie BUZZI^2,3^, Orly PETIPA NGA^4,5^, Cécile COUCHOUD^6^**, on behalf of the REIN registry

**Table 2** : Adjusted Hazard ratio of dialysis patients with their confidence intervals

| \| **Effect** \| **HR** \| **Lower cl** \| **Upper cl** \| \| --- \| --- \| --- \| --- \| \|  \|  \|  \|  \| \| **Age 0-20** \| 0,181 \| 0,074 \| 0,446 \| \| **Age 21-40** \| 0,719 \| 0,593 \| 0,871 \| \| **Age 61-80** \| 1,369 \| 1,282 \| 1,461 \| \| **Age 81+** \| 1,818 \| 1,695 \| 1,949 \| \| **BMI 0-18.5** \| 1,452 \| 1,337 \| 1,576 \| \| **BMI 25-30** \| 0,911 \| 0,875 \| 0,948 \| \| **BMI 30+** \| 0,883 \| 0,844 \| 0,923 \| \| **Male** \| 1,202 \| 1,160 \| 1,247 \| \| **Diabetes** \| 1,156 \| 1,115 \| 1,198 \| \| **Stroke** \| 1,038 \| 0,989 \| 1,090 \| \| **Others cardiovascular conditions*** \| 1,540 \| 1,482 \| 1,600 \| \| **Hepatic cirrhosis** \| 1,874 \| 1,718 \| 2,043 \| \| **Cancer** \| 1,403 \| 1,338 \| 1,472 \| \| **HIV/AIDS** \| 1,047 \| 0,814 \| 1,346 \| \| **HBV** \| 0,931 \| 0,743 \| 1,168 \| \| **HCV** \| 0,962 \| 0,807 \| 1,147 \| \| **Oral care** \| 0,552 \| 0,486 \| 0,524 \| \| **Waiting list** \| 0,266 \| 0,249 \| 0,285 \|   **Peritoneal dialysis** 1,446 1,367 1,530  **HD in emergency**  1,223 1,178 1,271 |
| --- | --- | --- | --- | --- | --- | --- | --- | --- | --- | --- | --- | --- | --- | --- | --- | --- | --- | --- | --- | --- | --- | --- | --- | --- | --- | --- | --- | --- | --- | --- | --- | --- | --- | --- | --- | --- | --- | --- | --- | --- | --- | --- | --- | --- | --- | --- | --- | --- | --- | --- | --- | --- | --- | --- | --- | --- | --- | --- | --- | --- | --- | --- | --- | --- | --- | --- | --- | --- | --- | --- | --- | --- | --- | --- | --- | --- | --- | --- | --- | --- |

*at least one of the following: coronary diseases, heart failure, rhythm disorders, lower limb arteritis, aortic aneurysm


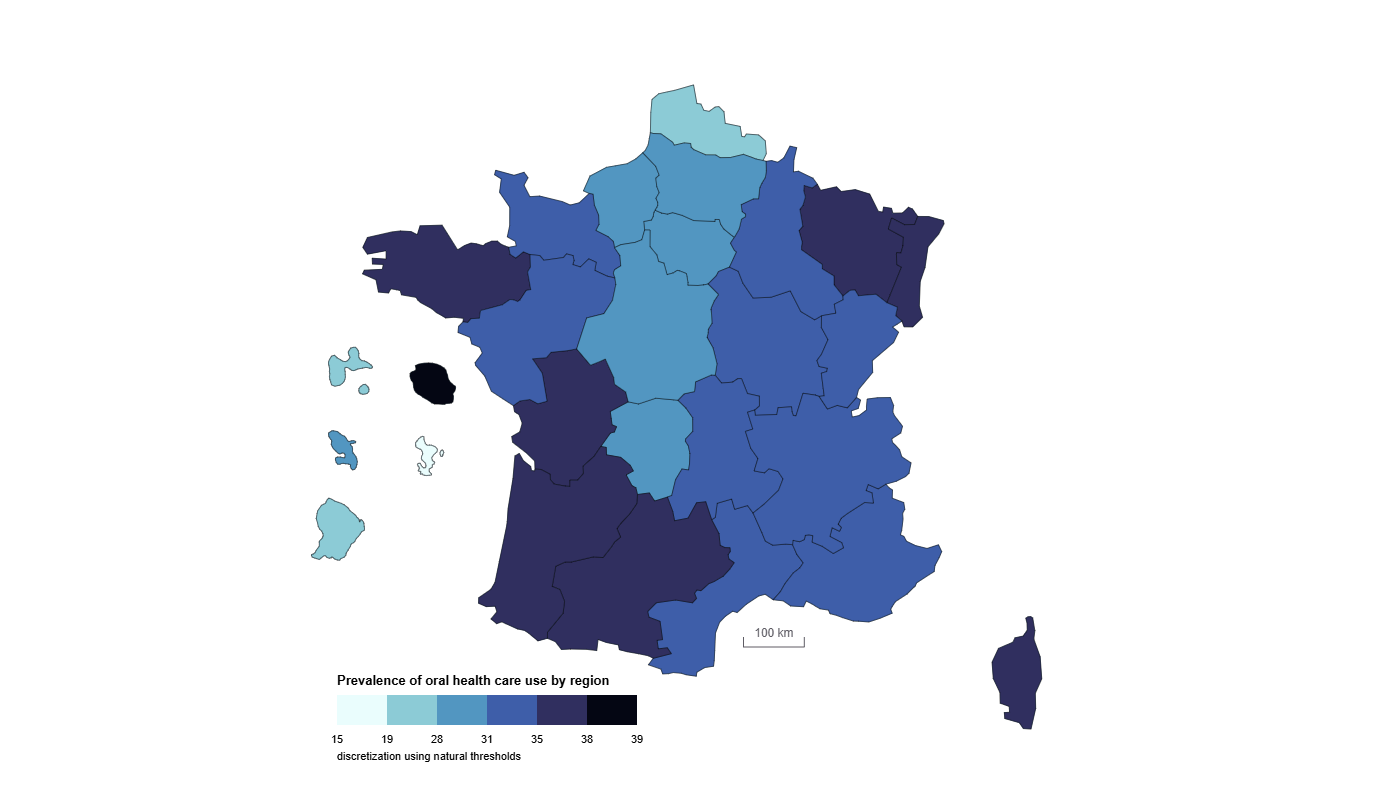


**Figure 1**: Regional distribution of oral care recourse of dialysis patients in France between 2015 and 2020


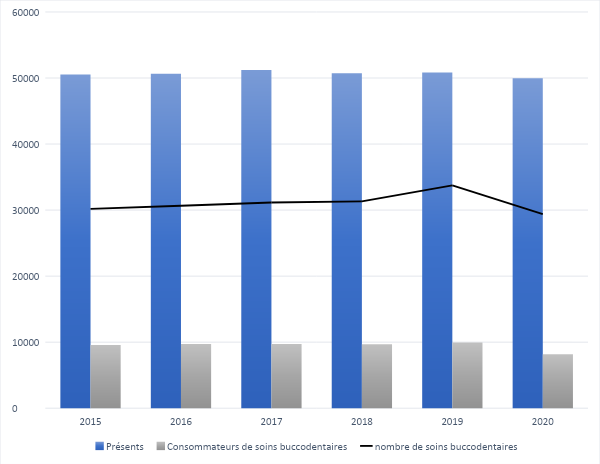



Dialysis patients

Oral care users

 number of oral care treatments

**Figure 2:** Annual evolution of oral care visits, oral care users and dialysis patients per year between 2015 and 2020 in France.
